# Comparison of patient-specific computational models vs. clinical follow-up, for adjacent segment disc degeneration and bone remodelling after spinal fusion

**DOI:** 10.1371/journal.pone.0200899

**Published:** 2018-08-30

**Authors:** Marc van Rijsbergen, Bert van Rietbergen, Veronique Barthelemy, Peter Eltes, Áron Lazáry, Damien Lacroix, Jérôme Noailly, Marie-Christine Ho Ba Tho, Wouter Wilson, Keita Ito

**Affiliations:** 1 Orthopaedic Biomechanics, Department of Biomedical Engineering, Eindhoven University of Technology, Eindhoven, the Netherlands; 2 National Center for Spinal Disorders, Buda Health Center, Budapest, Hungary; 3 INSIGNEO Institute for in silico Medicine, Department of Mechanical Engineering, University of Sheffield, Sheffield, United Kingdom; 4 BCN MedTech, Department of Information and Communication Technologies, Universitat Pompeu Fabra (UPF), Barcelona, Spain; 5 University of Technology of Compiègne, Compiègne, France; Universidad de Zaragoza, SPAIN

## Abstract

Spinal fusion is a standard surgical treatment for patients suffering from low back pain attributed to disc degeneration. However, results are somewhat variable and unpredictable. With fusion the kinematic behaviour of the spine is altered. Fusion and/or stabilizing implants carrying considerable load and prevent rotation of the fused segments. Associated with these changes, a risk for accelerated disc degeneration at the adjacent levels to fusion has been demonstrated. However, there is yet no method to predict the effect of fusion surgery on the adjacent tissue levels, i.e. bone and disc. The aim of this study was to develop a coupled and patient-specific mechanoregulated model to predict disc generation and changes in bone density after spinal fusion and to validate the results relative to patient follow-up data. To do so, a multiscale disc mechanoregulation adaptation framework was developed and coupled with a previously developed bone remodelling algorithm. This made it possible to determine extra cellular matrix changes in the intervertebral disc and bone density changes simultaneously based on changes in loading due to fusion surgery. It was shown that for 10 cases the predicted change in bone density and degeneration grade conforms reasonable well to clinical follow-up data. This approach helps us to understand the effect of surgical intervention on the adjacent tissue remodelling. Thereby, providing the first insight for a spine surgeon as to which patient could potentially be treated successfully by spinal fusion and in which patient has a high risk for adjacent tissue changes.

## Introduction

Almost everyone experiences low back pain during their lifetime [1,2]. This high prevalence and its relative morbidity contribute to its large socio-economic burden [2]. Although the exact cause of low back pain is often unclear, there is a strong association with intervertebral disc degeneration [3,4]. If conservative symptomatic treatment fails, surgical intervention is considered [5]. Spinal fusion is the standard surgical treatment, however, results are somewhat variable and unpredictable [6,7]. Moreover, it has been demonstrated that fusion is associated with a risk for accelerated disc degeneration at neighbouring levels, most likely due to the loss in flexibility at the fused level causing altered loading conditions at neighbouring levels [8,9,10,11]. Presently, there is some ambiguity in the literature concerning whether fusion is the cause for accelerated disc degeneration at adjacent levels. Some studies report accelerated disc degeneration while others do not (see e.g. [11]). A possible explanation for these conflicting conclusions is that the outcome of each study is strongly affected by patient-specific factors, such as spinal geometry, level of activity, osteoporosis, etc. This, in turn, would suggest that a patient-specific evaluation is needed to better select fusion patients.

Whereas fusion may directly change the loading conditions at neighbouring discs, it may also change loading conditions at neighbouring vertebrae. According to Wolff’s law, changes in loading will lead to changes in bone density and architecture, which, in turn, may also lead to local changes in disc loading at neighbouring levels. However, similar to the disc, also the reported findings of changes in bone mineral density (BMD) do not conform. Some researchers reported a decrease in BMD at the adjacent level [12] while others found an increase [13,14]. Possibly these differences reflect differences in loading of the vertebrae before and after the surgical intervention. This would suggest that changes in loading may be non-uniform, and, again, that a patient-specific evaluation is needed to better predict such changes.

The most advanced type of patient-specific models available nowadays are computer models based on clinical images. For the disc, several such models have been presented in the literature [15,16,17,18,19]. These models, however, typically represent only a specific stage of degeneration [20,15,16,17,18,19,21], mostly in a phenomenological manner and cannot account for continuous changes in the biochemical composition and structure of the disc over time during degeneration. Recently, a composition based FE model of an Inter Vertebral Disc (IVD) was developed [22]. In this model, the behaviour of the IVD is modelled by relating the local extra cellular matrix (ECM) composition and organization directly to its mechanical behaviour. Thus, the annulus fibrosus (AF) and nucleus pulposus (NP) properties depend directly on its constituent (water, fixed charged density, collagen and ground substance) material properties proportional to their content within the tissues. However, in order to become a predictive model, it should also account for changes in biochemical composition over time due to changes in cell activity. Several models have been developed to predict such changes in connective tissue cell activity and ECM based on mechanical loading conditions sensed by the cells [23]. Although such models have been mainly used and validated to explain fracture healing [24], they in general can also predict tissue differentiation processes in other situations. Presently, however, no model is available that couples such advanced composition-dependent constitutive models of the disc with mechanoregulated tissue differentiation models.

For bone, several remodelling theories based on Wolff’s law have been formulated to predict changes in bone density related to changes in mechanical loading [25]. It has been demonstrated that such models can successfully predict changes in bone density after total hip [26] and knee arthroplasty [27]. In the case of spinal fusion, however, the situation is more complicated since changes in overall loading for neighbouring segments are expected due to the reduced flexibility of the fused segment, but also changes in local load transfer due to degeneration of adjacent discs. To study such changes, a coupled analysis of bone remodelling and disc degeneration is required in combination with a whole spine model to predict changes in overall loading.

The aim of this study was to develop such a coupled and patient-specific mechanoregulated model to predict disc generation and changes in bone density after spinal fusion and to validate the results relative to patient follow-up data. Therefore, first, the constituent based FE model of an IVD, including vertebrae and ligaments [22], was extended to a mechanoregulated IVD model and it was demonstrated that a stable (steady-state) healthy IVD with proper tissue morphology was maintained under physiological loading. Next, the IVD model was extended with a previously developed bone remodelling theory [28] and extended to a patient-specific (pt-specific) algorithm. This was done by coupling it to a full lumbar spine FE model [29] as was developed in the framework of the EU-funded project MySpine. From this full lumbar spine model, pt-specific changes in loading due to fusion at the adjacent level were derived and subsequently, converted to the IVD model. By doing this, tissue adaptation at the adjacent level was simulated for both bone and IVD tissue. To demonstrate the clinical feasibility of this approach, patient-specific models were made for 10 patients that underwent fusion surgery and the predicted computational results were compared to clinical follow-up data.

## Material and method

First, the constituent based FE model of an IVD was extended to a mechanoregulated IVD model. To do so, a mechanoregulated tissue differentiation theory [23] was implemented, resulting in a stable healthy IVD that serves as starting point for IVD tissue adaptation simulations.

### Disc adaptation framework

#### Steady state tissue composition

**Fig 1:**
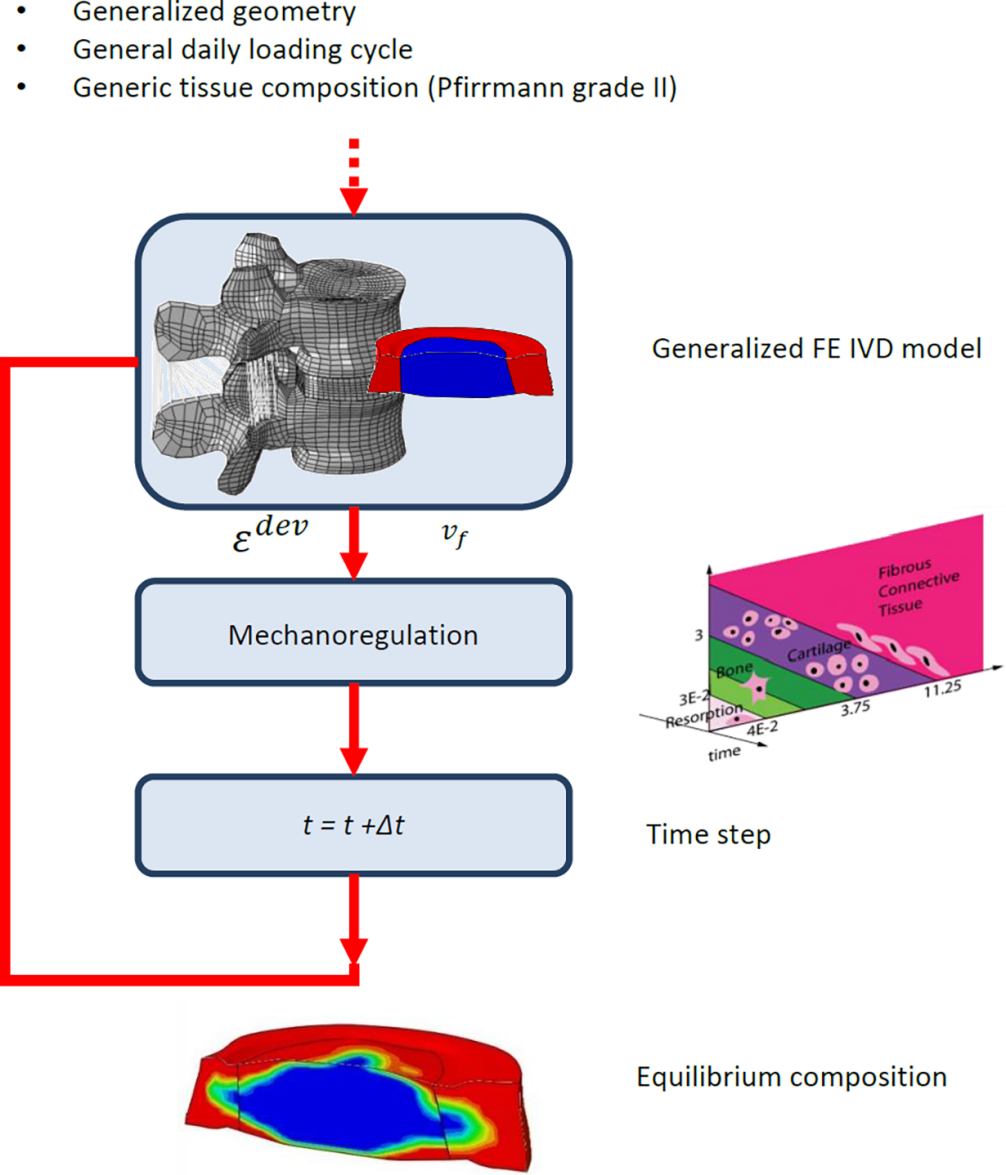
Flowchart describing the general method to simulate disc adaptation. To the generic IVD model a general daily loading pattern was applied, mimicking a loading cycle of a healthy generic person. Based on this loading pattern, per integration point (IP) a deviatoric shear strain and fluid velocity was obtained. These serve as input for the mechanoregulation algorithm and based on this algorithm, per IP, a preferred tissue phenotype is determined. The ECM content is adapted to this preferred phenotype with a fixed time step. After this adaptation step, the deviatoric shear strain and fluid velocity are re-determined and the whole cycle is repeated until no changes in ECM content were simulated.

The constituent based FE model of a healthy IVD [22] with generalized geometry was used as starting point (Fig 1). In this model, the NP and AF tissue properties are a function of their biochemical composition and microstructural organization and the mechanical behaviour is described according to [22] (Eq 1):

$$\sigma_{tot}=\frac{n_{s,0}}{J}((1-\sum_{i=1}^{tot_{f}}\rho_{c}^{i})\sigma_{nf}+(\sum_{i=1}^{tot_{f}}\rho_{c}^{i})\sigma_{f_{iso}}+\sum_{i=1}^{tot_{f}}\rho_{c}^{i}\sigma_{f}^{i})-\mu^{f}I-\Delta \pi I$$ (Eq 1)

where *n*_*s*,0_ is the initial solid volume fraction, *J* the determinant of the deformation tensor *F*,*σ*_*nf*_ the stress in the non-fibrillar matrix, $\sigma_{f_{iso}}$ the isotropic stress in the collagen fibres, $\sigma_{f}^{i}$ the tensile stress in the *i*^*th*^ fibril, $\rho_{c}^{i}$ the fibril density, *tot*_*f*_ the amount of fibrils, *μ*^*f*^ the water chemical potential, *I* the unit tensor and Δπ the osmotic pressure relative to the external physiological salt concentration (calculated based on the fixed charge density FCD). The distinction between IVD tissue properties, i.e. NP vs. AF, results from the differences in the distribution and organisation of the extracellular constituents (Table 1). For more detail, see [22].

**Table 1:** Input parameters disc model. Biochemical input parameters, for the IVD model [22]. The biochemical parameters are only used during the initial simulation of the disc adaptation. Thereafter, the mechanical stimulus determines per integration point what the content is.

	Nucleus pulposus	Annulus fibrosus
*n*_*s*,0_: initial solid volume fraction [% wet weight]	20	25
Collagen content ($\rho_{c}^{i}$) [% dry weight]	15	65
FCD [mEq/ml]	0.30	0.20

To simulate IVD tissue remodeling, the model was extended by defining the cells and corresponding ECM components per integration point (IP). Two types of cells were assumed to be present: fibroblasts and chondrocytes. The fibroblasts could initially be mainly found within the AF tissue, while the chondrocytes are responsible for maintaining the NP. Both types of cells maintain their respective phenotypic ECM, consisting of collagen and glycosaminoglycans (GAG). Collagen content is a direct input for the disc model [22], whereas GAG content is converted into fixed charge density (FCD). This was done by taking into account the molecular weight (MW) and negative charges per molecule of chondroitin sulfate (CS) and keratan sulfate (KS) present in fixed proportions as GAG molecules [30], as well as the fluid fraction (volume based) at each IP. Based on this new matrix content (collagen and GAG), the model was allowed to come to swelling equilibrium and thus, allowed to come to its preferred water content (self-determined).

The behaviour of each cell was determined by a mechanical stimulus (MS). The MS was determined by applying generic boundary conditions representing a normal daily loading cycle (axial compression, flexion, dynamic axial rotation and dynamic lateral bending, representing standing, sitting, periodic movements and walking respectively). Axial compression represents the action of external loads as well as muscle activation and is hence constantly present in almost all daily recreational and occupational activities. The magnitude of the compressive load (300 N and 30 N for day and night load, respectively) was based on in vivo studies during daily activities with upright posture [31,32,33,34,35]. In addition to this axial compressive load, three additional bending rotations [31,32,33,34,35] were applied (while the axial compressive load was still present): flexion (2°) in combination with a creep period of half an hour to represent sitting; dynamic lateral bending (1.5°) to represent walking; and dynamic axial rotation (0.9°) for periodic movements during various daily activities of life (both at 0.5 Hz). Per activity, the distributions of average deviatoric shear strain *ε*^*dev*^ (defined as $\varepsilon^{dev}=\frac{2}{3}\sqrt{{\left(\varepsilon_{1}-\varepsilon_{2}\right)}^{2}+{\left(\varepsilon_{2}-\varepsilon_{3}\right)}^{2}+{\left(\varepsilon_{1}-\varepsilon_{3}\right)}^{2}}$) and fluid velocity *v*_*f*_ were determined. A time average magnitude of each parameter per activity was used for a daily activity level, resulting in a MS *ψ* [23,36] (Eq 2)

$$\psi =\left(\begin{array}{l} \frac{\left(\varepsilon_{s\;\tan\;ding}^{dev}\cdot 5.5)+(\varepsilon_{walking}^{dev}\cdot 2)+(\varepsilon_{period\_mov}^{dev}\cdot 4.5)+(\varepsilon_{sitting}^{dev}\cdot 4\right)}{4.67}+\\ \frac{\left(v_{s\;\tan\;ding}^{f}\cdot 5.5)+(v_{walking}^{f}\cdot 2)+(v_{period\_mov}^{f}\cdot 4.5)+(v_{sitting}^{f}\cdot 4\right)}{3} \end{array}\right)*\frac{1}{16}$$ (Eq 2)

where the constants, i.e. 5.5 for standing, 2 for walking, 4.5 for periodic movements and 4 for sitting resp., represent the time weighted average per activity in hours. Finally, to determine the daily activity level, the summation of *ε*^*dev*^ and *v*_*f*_ was divided by 16 hours. The MS determines the preferred phenotype per integration point. The theory of Prendergast et al. [23,36] was adjusted to account for baseline residual strains in the unloaded disc (swelling-collagen tension equilibrium) [22]. This was based on the assumption that the original mechanoregulation theory did not take residual strains into account and that these strains are on the same order of magnitude as load-induced strains in IVD tissues where substantial osmotic swelling is balanced by collagen tensile strains. Initially, the threshold for strain and fluid velocity between the different preferred cell phenotypes was adjusted until the MS inside the geometrically defined nucleus region was mostly cartilage-favoured, i.e. the threshold between cartilage and fibrotic preferred phenotype was shifted in deviatoric strain from 11.25% to 14%; fluid velocity threshold was not adapted. Therefore, Eq 2 deviates from the original mechanoregulation theory [36], i.e. $\frac{\varepsilon^{dev}}{4.67}$ instead of $\frac{\varepsilon^{dev}}{3.75}$.

Dependent on the preferred phenotype, cells went into proliferation, differentiation or into apoptosis and produced or degraded matrix, according to (Eq 3).

$$\frac{\partial c_{i}}{\partial t}=f_{i}^{PROLIF}\left(\Psi \right)∙c_{1}∙(1-\frac{c_{i}}{c_{space}})-f_{i}^{DIFFER}\left(\Psi ,c\right)-f_{i}^{APOPT}\left(\Psi \right)∙c_{i}$$ (Eq 3)

in which *c*_*i*_ is the concentration of cell type i, i.e. chondrocytes or fibroblasts, $f_{i}^{PROLIF}$ is the proliferation rate of cell type i, *c*_*space*_ for the maximum allowed cell concentration minus the current concentration, $f_{i}^{DIFFER}$ for the differentiation rate of cell type i and $f_{i}^{APOPT}$ for the apoptosis rate of cell type i. The proliferation, differentiation and apoptosis rates for each cell type were constants and were either on or off, i.e. according to the preferred phenotype according to the MS. The different rates for the two cell types were previously calculated from experimental data collected in an extensive literature review [37] (Table 2).

**Table 2:** Cell behaviour. Proliferation differentiation and apoptosis rate of fibroblast and chondrocytes chondrocytes were previously calculated in normalized fashion from experimental data collected in an extensive literature review [37].

	$f_{i}^{PROLIF}$ (day ^*-1*^*)*	$f_{i}^{DIFFER}$ *(day* ^*-1*^*)*	$f_{i}^{APOPT}$ *(day* ^*-1*^*)*
Fibroblast	0.5	0.2	0.05
Chondrocyte	0.2	0.14	0.1

Production and degradation of matrix was modeled as cell based. Both cell types could produce and degrade collagen and GAG although the rates at which this is possible were different between the two cells. The rate, at which matrix was produced, was dependent on the MS and the current cell concentration (Eq 4).

$$\frac{\partial m_{i}}{\partial t}=f_{i}^{synth}\left(\Psi \right)∙c_{fb}∙\left(1-\frac{m_{i}}{m_{i}{}_{fb}}\right)-f_{i}^{degrad_{1}}\left(\Psi \right)∙c_{fb}-f_{i}^{degrad_{2}}∙c_{fb}∙\frac{m_{i}-m_{i_{fb}}}{m_{i_{fb}}}+f_{i}^{synth}\left(\Psi \right)∙c_{cc}∙\left(1-\frac{m_{i}}{m_{i_{cc}}}\right)-f_{i}^{degrad_{1}}\left(\Psi \right)∙c_{cc}-f_{i}^{degrad_{2}}∙c_{cc}\frac{m_{i}-m_{i_{cc}}}{m_{i_{cc}}}$$ (Eq 4)

These equations contained for both cell types several components: 1) a synthesis part $f_{i}^{synth}$ where *i* stands for GAG and collagen resp., in which the rate was dependent on cell type, with corresponding fixed rate (literature based), as well as the preferred target value, e.g. if the current amount of GAG is very small compared to the preferred amount, the synthesis rate is high; 2) two types of degradation: *degrad*_*1*_ is degradation of matrix by the non-preferred cell phenotype (literature based, Table 3), *degrad*_*2*_ is a degradation rate dependent on the current amount of matrix present compared to the preferred amount (fixed rate: 0.0011 (*day* ^*-1*^), independent of cell phenotype. Since no literature value was available for the latter, it was assumed to be in the same range as *degrad*_*1*_ rate. If the current amount was much higher than the preferred concentration, the *degrad*_*2*_ rate was higher. For example, when going from fibrous tissue to cartilage, chondrocytes produce collagen and GAG and fibroblast degrade their matrix, i.e. collagen and GAG. However, since the concentration of collagen is too high for cartilage, chondrocytes will also break down collagen. In addition to this, there is a natural turn-over by chondrocytes and fibroblast which continuously produce and degrade their own matrix. For healthy IVD tissue, i.e. both healthy NP and AF tissue, the half-life time of collagen is approx. 95 years [38] (0.00003 *day* ^*-1*^), the half-life time of GAG is 12 and 11.2 years for NP and AF tissue, respectively (0.00016 *day* ^*-1*^) [39]. To account for this, Eqs 4 and 5 were extended with a continuous production and degradation of GAG and collagen by the preferred cell type.

**Table 3:** Production and degradation rates of matrix components by fibroblast and chondrocytes [40,41,42,43]. The $m_{i_{fb}}$ represents the preferred matrix content of the fibroblast; $m_{i_{cc}}$ represents the preferred matrix content of chondrocytes.

	Matrix component	$m_{i_{fb}}$ resp. $m_{i_{cc}}$	$f_{i}^{synth}$ (day ^*-1*^*)*	*degrad*_*1*_*(day* ^*-1*^*)*
Fibroblast	Collagen	0.65	4.8 e-3	2.6 e-3
	GAG	0.35	0.85 e-3	0.45 e-3
Chondrocyte	Collagen	0.15	3.1 e-3	1 e-3
	GAG	0.85	7.7 e-3	2 e-3

The process of tissue adaptation then was repeated several times until no more changes in disc composition were found, i.e. reaching the equilibrium state. It was assumed though that the orientation of the collagen fibres in the AF will not change due to adaptation. This equilibrium state was used as starting point to investigate the effect of surgical intervention. Due to surgical intervention the boundary conditions (BC) change, caused by a change in kinematic behaviour, resulting in a different MS per integration point (IP) and subsequently in a different ECM composition per IP (Fig 1). An explicit time integration scheme is implemented to simulate the changes in disc properties over time (time step of one day).

#### Patient-specific implementation

**Fig 2:**
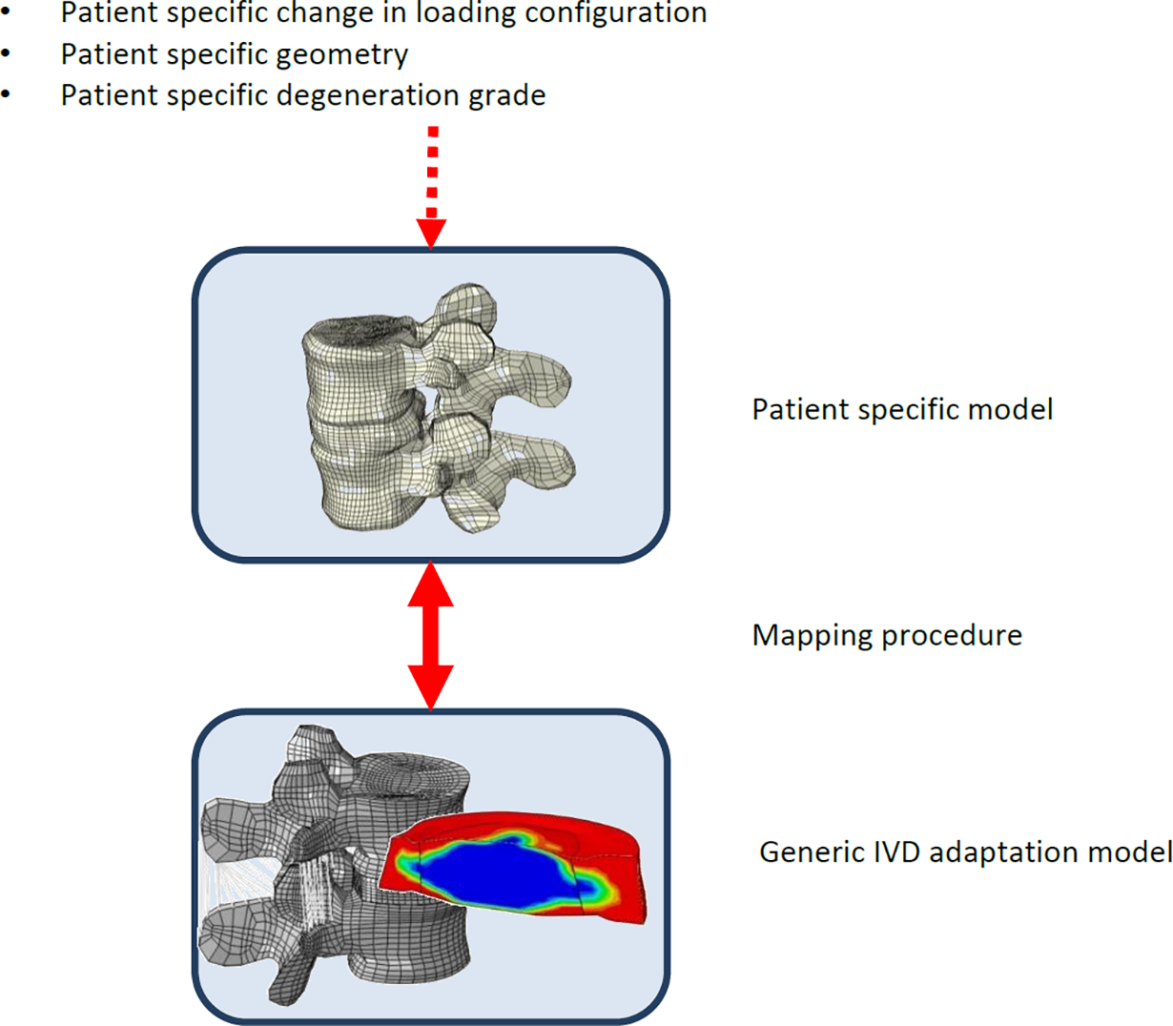
Patient-specific disc adaptation implementation. To make predictions for a patient, first a patient-specific model is generated, including the patient specific composition, geometry and changes in loading conditions. These properties are transferred to the standard model using a mapping procedure. With this procedure, changes in any of the parameters are translated to equivalent changes in parameters used for the standard model. The results of the adaptation simulation are mapped back to the patient model.

An FE model of the patient disc was created based on the geometry obtained from the patient MR-scan. The composition of the disc was based on the Pfirrmann grade using the same MR-scan. Since the calculation of the equilibrium situation as described above is very time consuming and requires knowledge of patient-specific loading conditions, it was not possible to do this for each patient. To be able to predict patient-specific changes, an alternative approach was used. With this approach, the patient-specific disc model was used only to calculate the element composition: the initial solid volume fraction *n*_*s*,0_, the fibril density $\rho_{c}^{i}$, and the fixed charge density FCD. These parameters were then mapped to the mechanoregulated disc with generalized geometry (Fig 2). This mapping is possible because all patient models are derived from a fixed mesh template that is morphed to the patient based on anatomical landmarks [44,45]. Since all elements thus have the same anatomical position, it is possible to transfer information from the patient-specific to the generalized model. The simulation of the mechanoregulation process then is performed using the generalised model, to predict changes in the disc composition, and the results are mapped back to the patient-specific model where they were used to calculate the composition-dependent material properties. The sequence of loading conditions representing a normal daily loading cycle was applied again, but scaled in magnitude according to the change in loading obtained from the lumbar spine model, before and after fusion. Directly after applying the adapted loading configuration due to fusion surgery to the model, the tissue changes were expected to be the largest. Therefore, the tissues were allowed to remodel for 3 months before the model was re-run to determine the new mechanical stimulus. After the first year, smaller changes were expected as the largest adaptation took place during the first year, the time step was set to 6 months.

### Bone adaptation framework

To account for changes in the adjacent vertebra, i.e. changes in bone stiffness, the model was extended with a previously developed bone remodelling theory [28]. This theory is a homogenized version of an earlier microstructural bone remodelling theory [46,47] and predicts the bone density as a function of mechanical loading, quantified by the strain energy density (SED), and parameters related to cell-level activity and microstructure. Similar to the disc adaptation theory, the actual bone adaptation takes place at the level of the cells and is assumed to be regulated by the tissue-level SED at the surface of the bone tissue *S*_*T*_ as sensed by the cells. Based on this mechanical signal, the osteocyte sensitivity μ and the osteoblast bone formation rate τ, the amount of newly formed bone is calculated. Also at this level, osteoclast cells were assumed to remove bone tissue at a constant speed determined by the osteoclast resorption chance *f*_*ocl*_ and resorption volume *V*_*res*_. Based on these parameters and the available bone surface per volume *BS/TV*, of which a fraction *α* was assumed to be available for bone remodelling, modelled as cylindrical structures, the bone resorption rate is calculated. By integrating these rates over the bone volume, the total change in bone density *BV/TV* (dimensionless, range 0–1.0) then is calculated as:

$$\frac{d(BV/TV)}{dt}=Const_{SED}\cdot \alpha \cdot \left(BS/TV\right)$$ (Eq 5)

where *Const*_*SED*_ is defined as

$$Const_{SED}=(\tau \mu S_{T}-f_{ocl}V_{res})$$ (Eq 6)

As with the disc adaptation, an explicit time integration using fixed time step of one day is used to predict the changes in bone density over time.

#### Patient-specific implementation

**Fig 3:**
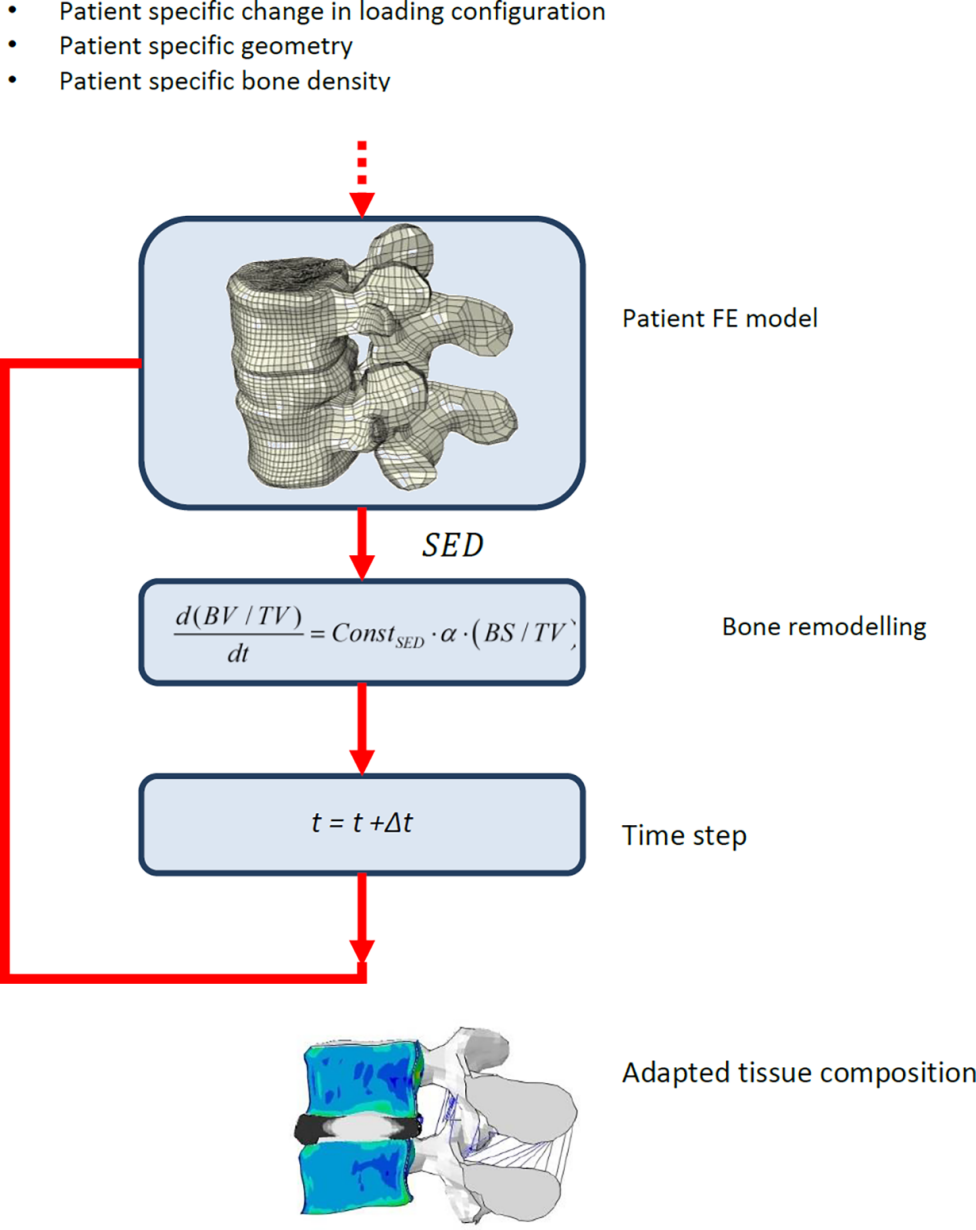
Patient-specific bone adaptation implementation. From CT scan of a patient, bone density and the geometry are obtained. From the full lumbar model patent-specific boundary conditions and changes in these boundary conditions due to fusion are obtained. The patient-specific FE model is used to determine the organ level load distribution. Using the bone remodelling theory, this is translated to a change in the density distribution.

For the patient-specific implementation, the bone geometry and density distribution were based on the direct pre-operative patient CT scan. Whereas the bone remodelling simulation was performed for the full vertebrae, results will focus on changes in the bone density of the vertebral core. Similar as for the disc, applying the remodelling equations (Eqs 5 and 6) would typically require a time consuming tuning procedure. This could be solved by using a mapping procedure, similar to that used for the disc. A more elegant solution in this case, however, is to use the pre-operative state as the reference state and to take the changes before and after the operation as the driving force for remodelling (‘site specific‘ bone remodelling [25]) (Fig 3). The bone remodelling theory therefore was modified to enable the prediction of changes in bone density as they relate to changes in bone loading by defining the stimulus as the relative difference in the SED distribution after and before the fusion operation:

$$Const_{SED}=(\tau \mu SED_{post-op}-f_{ocl}V_{res})-(\tau \mu SED_{ref}-f_{ocl}V_{res})$$ (Eq 7)

The pre-operative SED, or SED_ref_, distribution was calculated based on the direct pre-operative density distribution as obtained from the patient CT scan using loading conditions obtained from the full lumbar model (see below) representing the spine before the operation. The post-operative SED was calculated using the bone density distribution predicted by the remodelling theory using loading conditions obtained from the full lumbar model with fused disc. If both are similar, no remodelling will occur, i.e. Eq 7 and Eq 5 will be zero resp. Directly after applying the adapted loading configuration resulting from fusion surgery, the tissue changes were expected to be the largest. Therefore, the tissues were allowed to remodel for 3 months before the model was re-run to determine the new mechanical stimulus. After the first year, smaller changes were expected as the largest adaptation took place during the first year, the time step was set to 6 months. For the bone remodelling only the compressive load case was considered since this was found to be the most dominant loading mode and inclusion of other loading modes did not change the results much.

In order to obtain realistic time-dependent bone remodelling patterns, some of the bone remodelling parameters were tuned. For this tuning, the bone density of one specific patient (patient #10, see below) was used as input. The remodelling process then was simulated using different values for the remodelling parameters until good agreement was achieved between the predicted 2-years results and the 2-years results obtained from the CT-scan. The values of the material parameters used is listed in Table 4.

**Table 4:** Bone remodelling parameters. The bone remodelling parameters as defined in the theory of Colloca et al. [28]. The bone formation rate *τ*, osteocyte mechanosensitivity *μ* and resorption volume per cavity v_res_ were fitted, marked with ** see below, other parameters based on Colloca et al. [28].

Parameter		Value	Unit
Bone formation rate	τ	475**	[μm^3^/(nmol·day)]
Osteocyte mechanosensitivity	μ	1**	[nmol/((MPa/s)·μm^2^)]
Osteoclast activation frequency	*f*_*ocl*_	0.03	[1/(day· mm^2^)]
Resorption volume per cavity	*V*_*res*_	5.6.10^−5^	[mm^3^]
Bone specific surface fraction	α	0.5**	[–]

### Lumbar model

**Fig 4:**
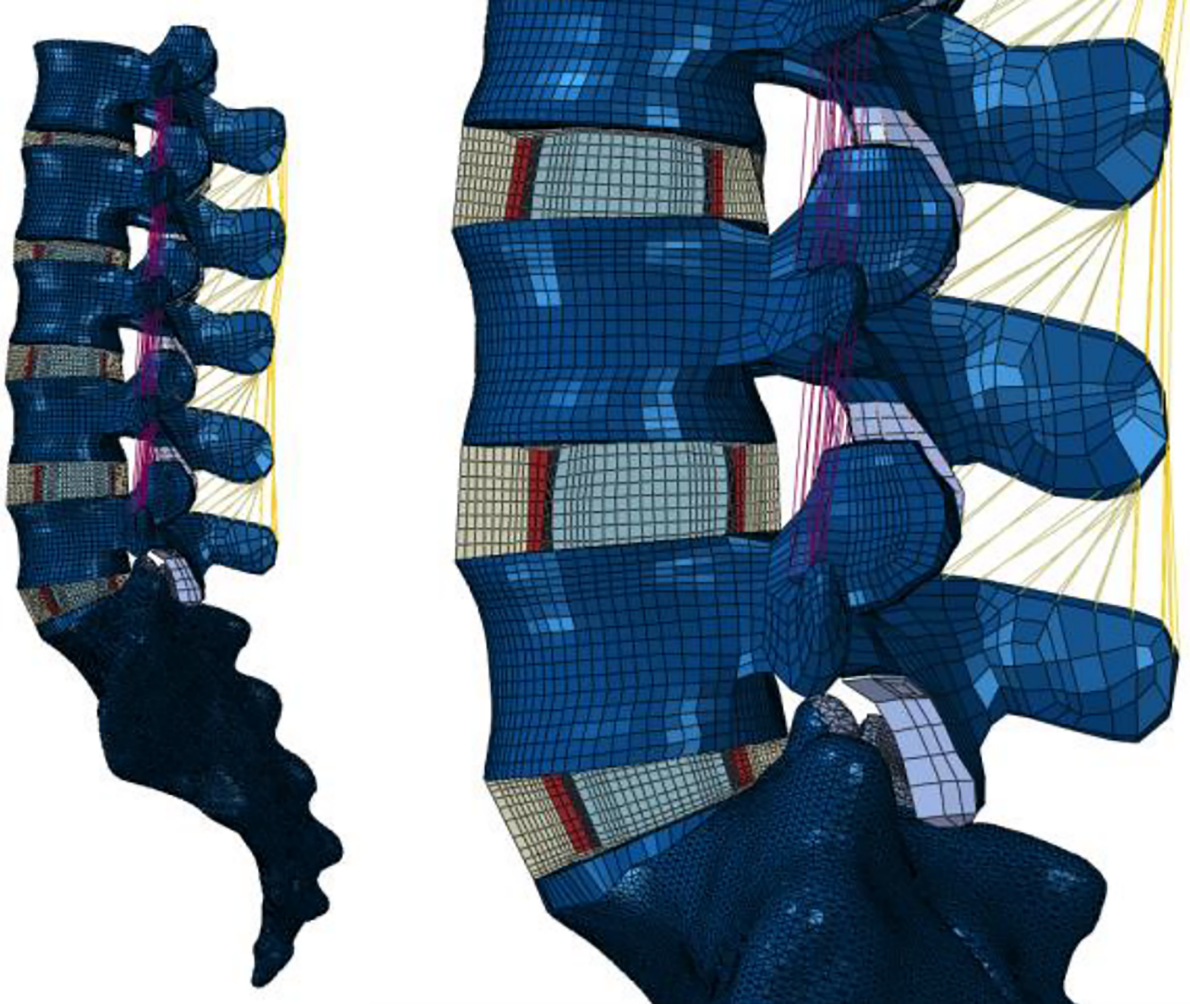
Example of lumbar model. An example of a lumbar FE model. The model contains vertebrae, discs and ligaments form L1 till S1.

The lumbar model is an FE model representing the full lumbar part (L1 –S1) of the spine [29] (Fig 4). This model, which includes vertebrae, discs and ligament, is generated from patient CT and MR scans and has a patient-specific geometry of both the vertebrae and discs [44,45]. Each vertebra was meshed with around 40,000 and each disc with around 20,000 8-node hexahedron elements, such that the total number of elements was around 300,000. Typical element size was between 1.5 and 2.5 mm.

The cortical bone and bony endplates were represented by a structural mesh layer at the periphery of the vertebral body models. The thickness of this layer varied depending on the location, i.e. middle, cranial/caudal, anterior or posterior cortical bone, and middle or peripheral bony endplate anterior posterior, and local thickness values were defined from direct measurements on histological cuts [48]. The material properties were chosen linear elastic, with a Young's modulus of 12 GPa in the axial direction and 8 GP in the transversal direction for the cortical bone and a Young's modulus of 250 MPa in the axial direction and 140 MPa in the transversal direction for the cancellous bone [16]. The AF and the NP were both modelled as poro-hyperelastic materials [29]; different to what is used for disc adaptation. The total stress tensor *σ* caused by external loads was the superimposition of the porous solid stress and the fluid pore pressure, that were respectively derived from a strain energy density function *W*, and Darcy's law [29].

$$W=\frac{G}{2}\left(I_{1}-3\right)+\frac{K}{2}{\left(J-1\right)}^{2}+W_{ani}$$ (Eq 8)

$$\underline{\underline{\sigma }}=\frac{1}{J}\frac{\partial W}{\partial \underline{\underline{F}}}{\underline{\underline{F}}}^{T}-p\underline{\underline{I}}$$ (Eq 9)

$${\underline{u}}_{f}\phi =\underline{\underline{k}}\cdot \underline{\nabla p}$$ (Eq 10)

where G and K are the shear and bulk modulus respectively of the porous solid (drained), *J* the determinant of the deformation tensor *F*, *I*_1_ the first strain invariant, *I* is the second order unit tensor, *u*_*f*_ the pore fluid velocity and *ϕ* and *k* are the porosity and hydraulic permeability tensor resp. The term *W*_*ANI*_ is an anisotropic strain energy density term, different from zero only for the AF. For more details, see Malandrino et al. [29].

The model included the seven major ligaments (anterior longitudinal, posterior longitudinal, capsular, supraspinous, ligamentum flavum, interspinous and intertransverse ligaments) as described before [22]. In summary, the ligaments, except for the posterior longitudinal ligament (PLL), were modelled with truss elements with an exponential stress-strain relationship for the toe region, followed by a linear behavior [16] as described by

$$\begin{array}{l} S=AE^{B}\\ S=CE+D \end{array}$$

where *S* is the 2nd Piola-Kirchhoff stress, *E* the Green strain and *A*, *B*, *C* and *D* four constants defined in Noailly et al. [49]. The PLL is not only attached to the vertebrae but also to the annulus. Thus, it was modified to match the AF posterior shape, and modelled with surface fibre-reinforced elements, tied to the edge of the vertebrae and the posterior annulus. The material properties of the rebars, i.e. the reinforcing fibres, were hypoelastic similar to the other ligaments. The articular facet surfaces were modelled with surface-to-surface frictionless contact. A similar model of a similar motion segment, but with a generic geometry, was validated earlier relative to experimental measurements and provided a good representation of the time-dependent decrease of the reaction forces, the range of motion for applied moments and facet forces [22].

Load boundary conditions, i.e. compressive forces and flexion moments, based on the patients’ weight and height, respectively, were simulated. Resultant forces (follower loads) and forward bending moments were applied at the centre of the cranial endplates, according to the musculoskeletal model calculations and anthropometry-dependent interpolations reported by Han et al [50]. In flexion, the effects of the imposed moment were always analyzed at 12^o^ of calculated rotation. The lumbar spine model was subsequently used to investigate the resulting load conditions per segment with and without simulated fusion. These local loads were used to calculate the percentage change in loading for the segment adjacent to fusion, which determined the proportion by which the daily loading cycle should be adjusted for the disc adaptation model, so as to reflect the mechanical effect of the fusion simulated patient-specifically.

### Integrated bone–disc adaptation

To study the effect of fusion surgery on both bone and disc simultaneously, the two algorithms were integrated into the same framework. Bone remodelling is assumed to be driven by the magnitude of the applied load [51], i.e. the magnitude of a peak load, while disc adaptation is assumed to be dependent on the average load distribution during normal daily activity (see disc adaptation framework description).

**Fig 5:**
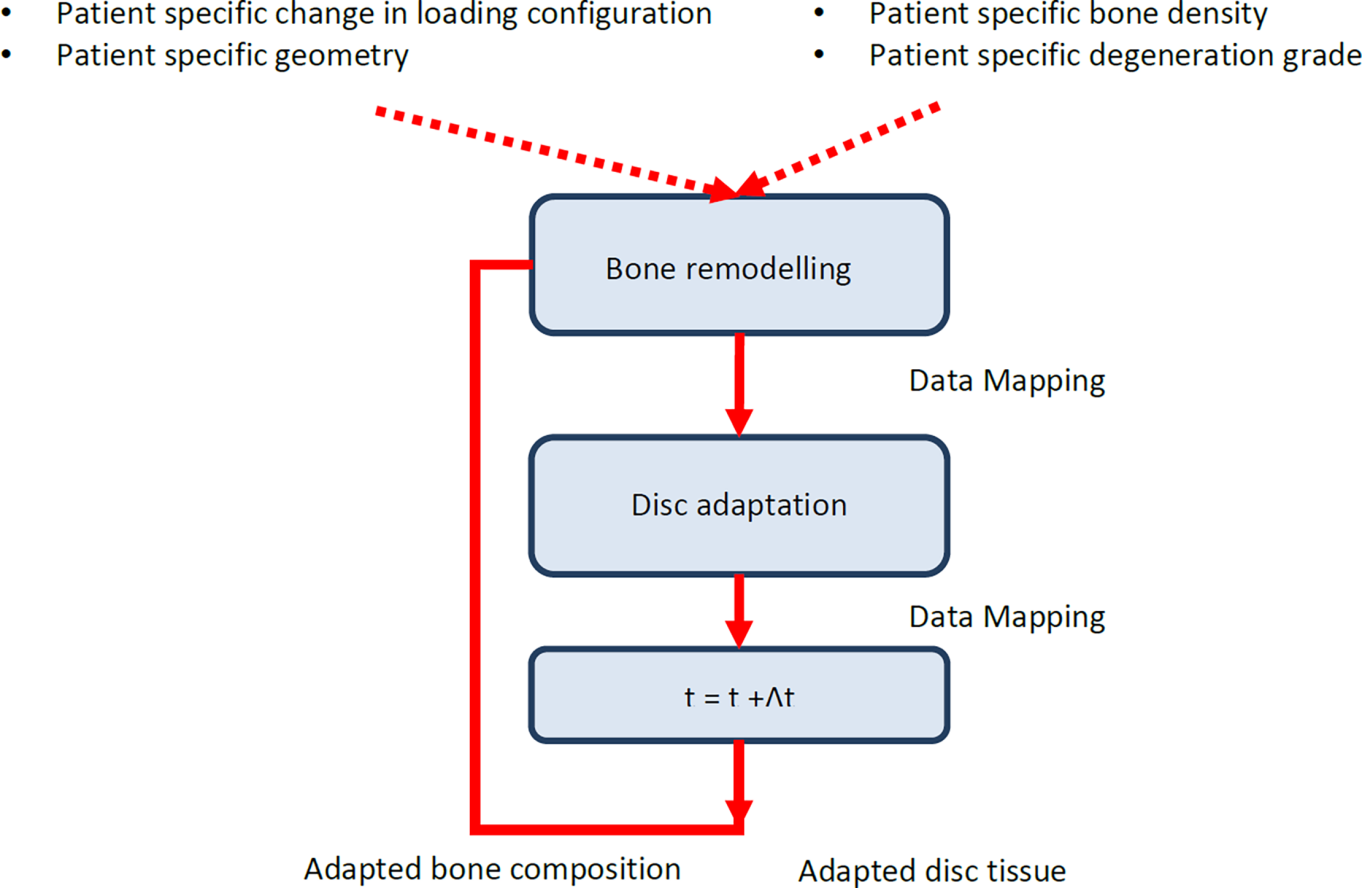
Coupling bone and disc adaptation simulation. In the coupled model, the bone remodelling and the disc adaptation algorithms are combined to represent the bone remodelling and disc adaptation due to intervention. Starting with the bone remodelling, a new density distribution is calculated that is mapped to the disc adaptation model. Changes in disc composition are calculated and are subsequently mapped back to the patient-specific model. Each module is explained in detail above.

Since bone remodelling occurs much faster than disc degeneration (order of months versus order of years), the simulation of bone remodelling requires much smaller time steps than that of disc adaptation. Using a fully coupled approach would require the evaluation of both the stresses/strains in the disc and in the bone at each time step. Since evaluating the average stresses/strains in the disc is very computationally expensive, and since the changes in disc composition would be minor for such small time steps, a semi-coupled approach was used instead. With this approach, the two processes where separated and performed in series to each other once for each larger time step, i.e. first bone remodelling followed by disc adaptation (Fig 5). Within each larger time step, bone remodelling simulations were allowed to come to their temporary steady state; as disc adaptation is a slower process, the adaptation was simulated in a fixed time frame not necessarily resulting in steady state. Before tissue adaptation was simulated (both disc and bone), the disc was allowed to come to mechanical steady-state by applying three preconditioning loading cycles consisting of an axial compression load [31,32,33,34,35] followed by a creep period, similar as described in the disc adaptation framework. This precondition mimics the action of external loads as well as muscle activation.

### Patient study

**Fig 6:**
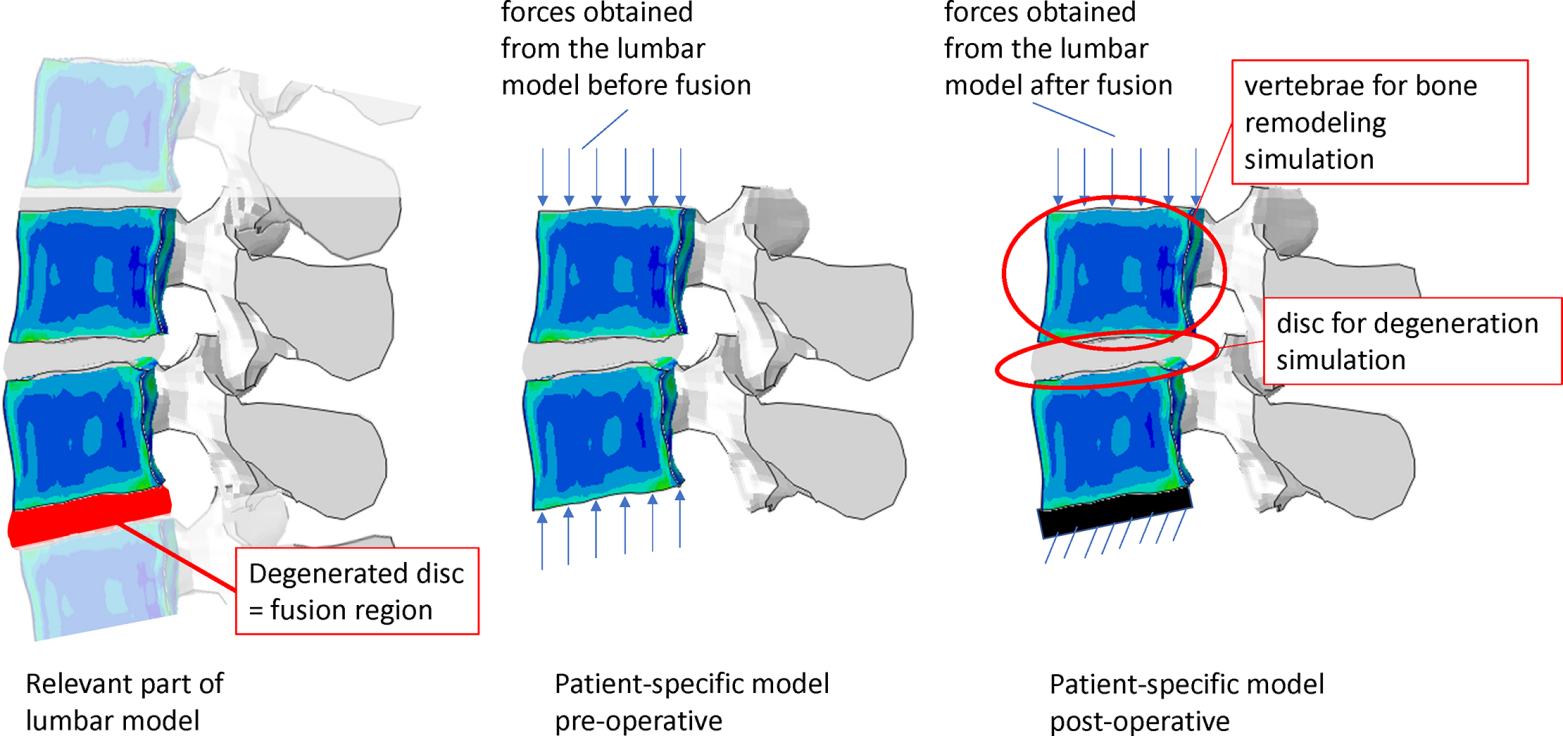
Overview of generating the models for the patient study. From the lumbar model the two vertebrae neighbouring the degenerated disc and the neighbouring disc are extracted as the ‘patient-specific’ model. Also the boundary conditions representing the pre-operative loading are extracted from the lumbar model. Following, the fusion surgery was simulated and new boundary conditions were calculated. For these new boundary conditions, changes in the disc and the vertebrae neighbouring the operated region were calculated.

To demonstrate the feasibility of this described generic framework to investigate changes in adjacent tissue after a fusion, it was applied to the data of 10 patients (pt) suffering from low back pain. The original database was built up in the EU-funded MySpine project and contained the prospectively collected clinical and imaging data of 192 patients treated at the National Center for Spinal Disorders according to the national guidelines and institutional protocols. This retrospective study was based on a subset of the database and approved by the Scientific and Research Ethics Committee of the Hungarian Medical Research Council (751/PI/2010). Patients with known metabolic bone disease were excluded from the study cohort. This cohort consisted of young adults (mean age: 38 years) where osteoporosis is rare. The cohort did not contain patients with clinically poor bone quality. Based on CT and MRI data, pt-specific FE meshes of the lumbar spine were reconstructed [29,44,45]. Out of these 192 cases, 27 patient cases underwent a monosegmental lumbar fusion. For 10 cases (7/10 male; mean age at surgery 38 years; 5/10 fusion at L4-L5, 4/10 fusion at L5-S1, 1/10 L3-L4), the lumbar spine model [29] could successfully solve all applied loading configurations (axial compression and flexion for the pre-surgical situation and after fusion treatment resp.). For these cases, the effect of fusion surgery on the adjacent disc and vertebra was simulated and compared to the clinical follow-up data (Fig 6).

### Outcome measures

Changes in bone density of the vertebra and changes in ECM at the adjacent level were compared to clinical follow-up (FU) data 2 years post-surgery. To compare the predicted bone densities, the simulated bone density value per location was compared to the clinically observed bone density at the same location 2 years post-surgery. This was done for the vertebra adjacent to the fusion level, e.g. when level L4-L5 was fused, the predicted and clinical densities of vertebra L3 were compared to each other. To do so, the same mapping and morphing procedure as to determine the initial geometry and bone density [44,45] was used for the 2 year FU data. To reduce the effects of noise in the CT scan and to obtain more meaningful bone density values, the element bone density was evaluated for a spherical region with a radius of 2 mm with its centre at the element centre [52]. The calculated density then was assigned to all integration points of the element. As elements were typically smaller than 2 mm, this resulted in overlapping evaluation regions when calculating densities of neighbouring elements, which naturally smoothens the density distribution. This was done for both simulated and clinical FU data. Since the reproducibility of the results depends on the reproducibility of the meshing algorithm, a small sub-study was performed to investigate errors that can be expected due to meshing errors. In this sub-study, meshes for four patients were generated three times, and the density distribution was calculated. Based on these results it was concluded that the mean detectable change (MDC) per element was 1.9%, indicating that changes less than this value cannot be detected. At the periosteal boundary of the vertebrae, however, a much lower reproducibility was found. This was due to the fact that the meshes were generated by segmenting the vertebrae at a rather low density, thus to be sure all bone was meshed. As a consequence of this, however, elements at the periosteal side sometimes were just outside of the bone region, showing a very low density. This did not affect the results of the stiffness or the remodelling though. The similarity between the simulation and FU data was expressed by a correlation coefficient, i.e. the bone density in each element at the adjacent level of the fusion was compared to the density in the same element in the simulation. In order to have a clinically relevant outcome for the disc changes, the relative changes in ECM content were translated into a change in pt-specific degeneration grade for the overall IVD based on tissue water content. The relative change in ECM content between different degeneration grades has been well studied in literature [53,54] and the best predictor of a change in degeneration grade is water content within the tissue [53]. As in the IVD model the water content is based on the equilibrium between collagen tension and GAG content-based osmotic swelling, changes in collagen and GAG content in IVD tissue lead to a change in water content. Due to the used modelling approach this predicted change in water content is a relative change. Prior to treatment, for each patient, the grade of degeneration was known, and thus the water content inside the IVD tissue [53]. After adaptive simulation, the calculated relative change in water content was translated to an absolute water content, based on the prior known pt-specific water content and the simulated change (grade dependent, based on literature [53], leading to the pt-specific change in water content and corresponding pt-specific change in grade. This was compared to clinical scored degeneration grade per patient 2 years post-surgery. Three randomly selected cases (#4, #6 and #9) were simulated up till 10 years post-surgery to study the effect of fusion on both adjacent vertebra and IVD at a longer time span. Although these results cannot be compared to clinical data, it shows the potential of the developed framework.

## Results

### Disc steady state

**Fig 7:**
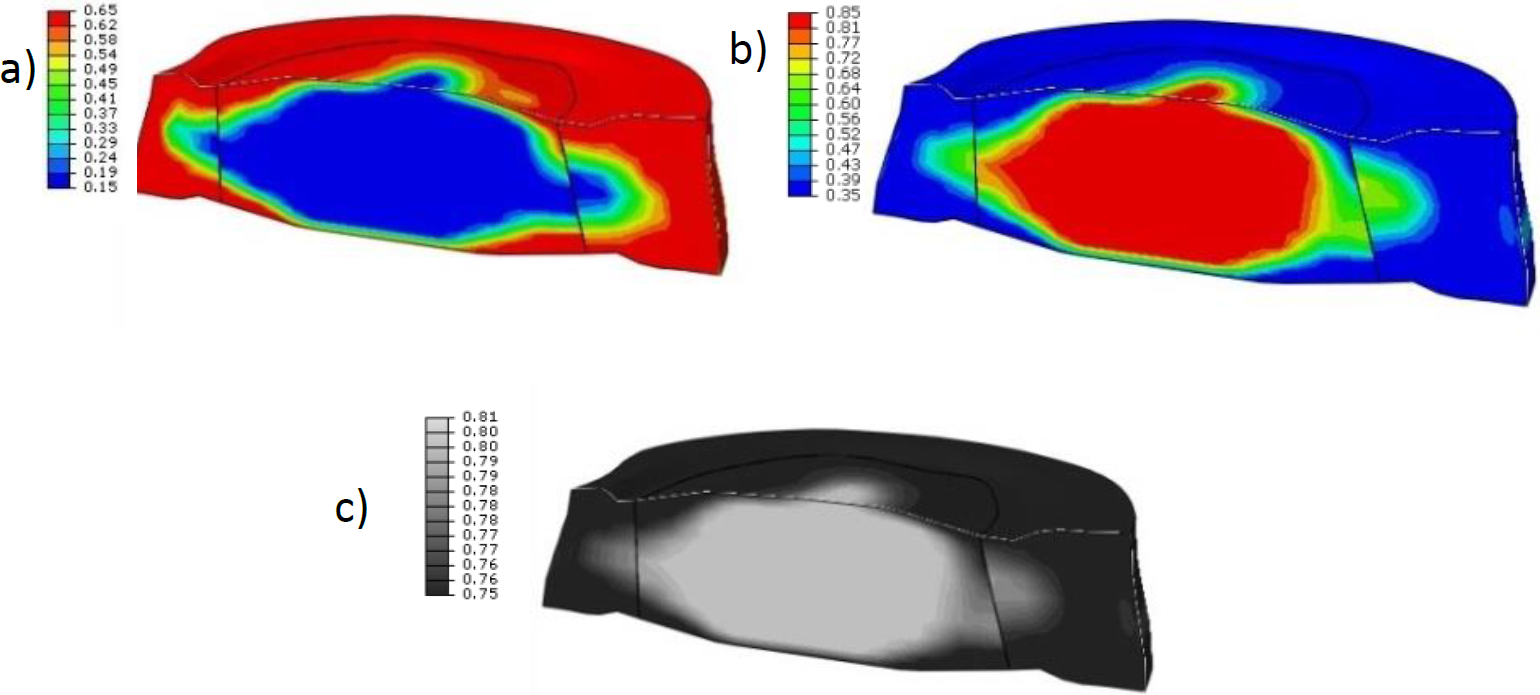
Extra cellular matrix content of a healthy disc. Cross-sections of a steady state disc with (a) collagen (b) GAG and (c) water (relative amount of content). In the centre of the disc, a region with low collagen (blue) and high GAG (red) and water content is found, similar to cartilage tissue, i.e. NP. Towards the outer shape of the disc, regions with high collagen and low GAG and water content are observed, i.e. AF tissue. In between these regions, a mixture of both tissues is observed, indicating the transition zone between both tissues. The black lines indicate the borders between the original geometrically defined NP and AF region.

To verify the adaptation method and to determine a steady-state IVD with corresponding matrix content and cell density spatial distribution, it was assumed that the adaptation changes for physiological loads should result in a steady-state IVD with tissue morphologies of a healthy normal IVD. After adaptation to steady state, in the centre of the IVD, a large region was present where cartilage tissue had formed. Circumferentially in the outer regions of the IVD, steady-state fibrous tissue had formed with corresponding matrix components. In between these two regions, particularly at posterior location of the IVD, a mixed tissue is present (Fig 7).

### Pt-specific simulations

#### Bone adaptation

**Fig 8:**
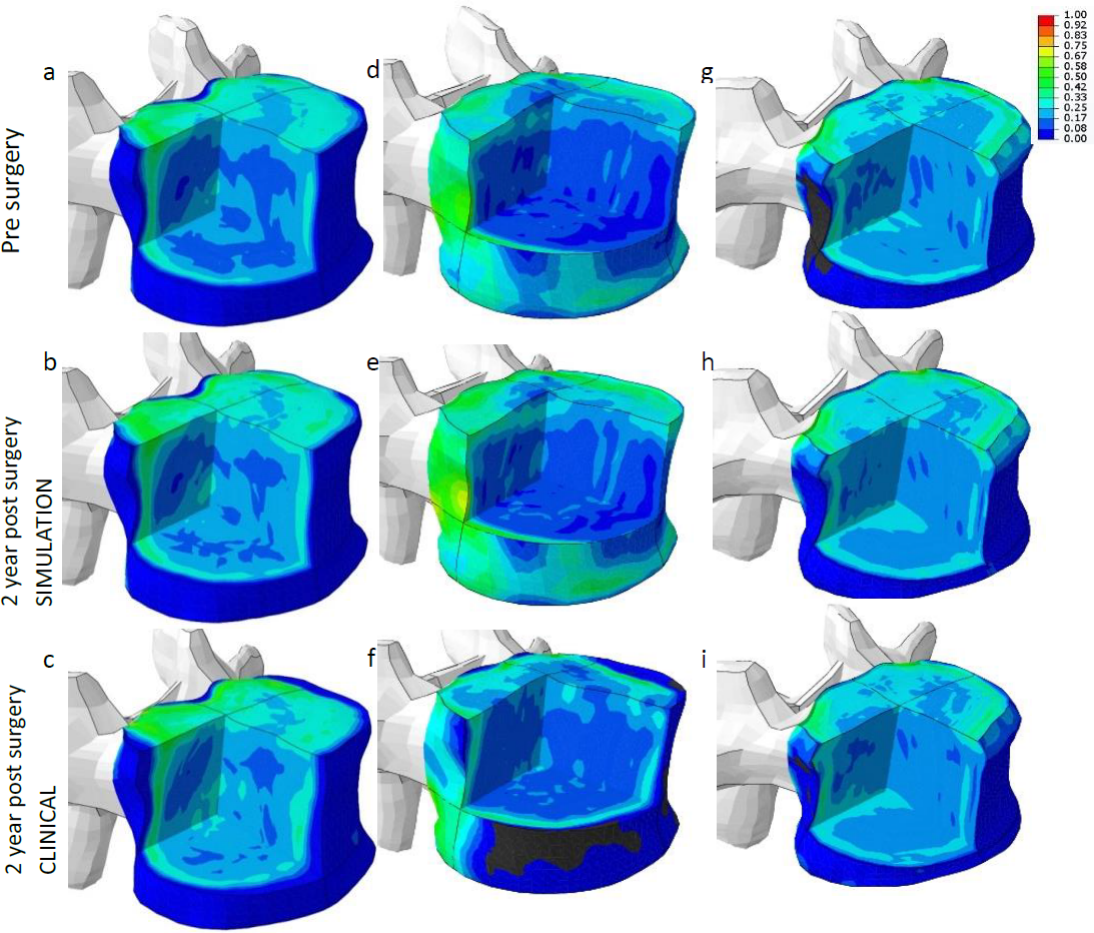
Examples of predicted vs clinical bone changes. Examples of graphical representation of bone density (BV/TV) as predicted 2 years post-surgery (b, e and h) and measured at the same time point (c, f and i), compared to pre surgery (a, d and g). a, b and c correspond to patient #1, d, e and f to patient #7, g, h and i to patient #10. A black colour indicates a density of 0. Note that the dark-blue appearance at the cortical shell is because the mesh boundaries were taken slightly larger than the vertebral size. As a result, the outer layer of elements can have a very low density.

In general, modest changes in the adjacent tissues were found, both in bone and IVD tissue. For bone, the predicted bone densities were in fair to good agreement with the clinical FU data (Table 5, Fig 8). The low density cortical appearance of the vertebrae in Fig 8 relates to the fact that elements at the most periosteal locations can be outside of the vertebral body. As mentioned earlier, however, this will not affect the results. The fact that these elements in some cases were just outside and some other case just inside the bone, however, did considerably reduce the correlations as calculated in Table 5. Note, in this section, the visual results of bone remodelling are visualized on the pt-specific adjacent vertebra only, i.e. the caudal vertebra of the segment adjacent to the fusion without disc.

**Table 5:** Correlation bone simulation. Correlation between predicted bone density (BV/TV) vs clinical FU bone density. Case #10 was used to fit the remodelling parameters, resulting in a very high correlation.

Case number	#1	#2	#3	#4	#5	#6	#7	#8	#9	#10
Correlation coefficient (r)	0.81	0.73	0.69	0.91	0.80	0.77	0.61	0.76	0.77	0.97

#### Disc adaptation

**Fig 9:**
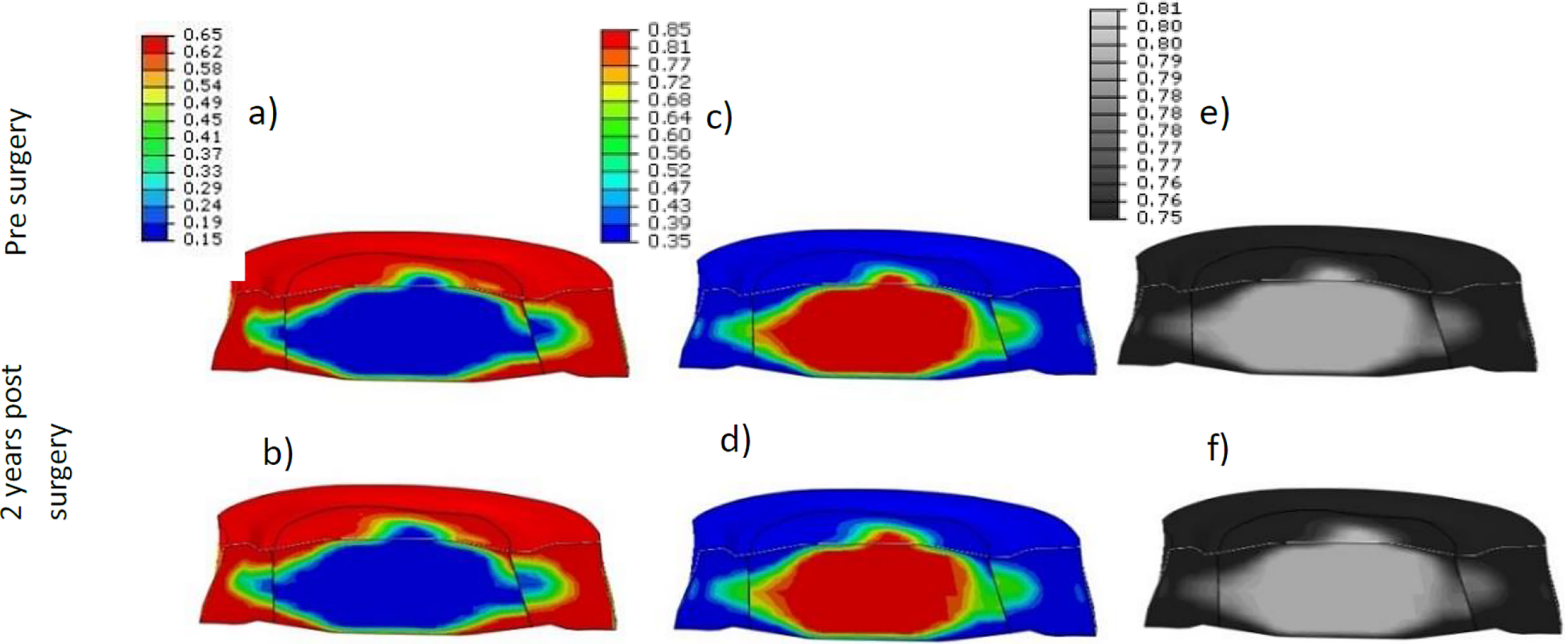
Typical extra cellular matrix changes. a) Start collagen content and predicted collagen content 2 years after fusion (b). c) and d) Change in GAG content for 2 year simulation due to fusion. e) and f) Changes in water content in the disc for 2 year simulation. For visualization purposes, only the disc is shown. Predicted changes are for case #9 and serve as example of the outcome for the other 9 cases.

As for the adjacent disc, in all patients, only modest changes in biochemical content were predicted by the simulation. When looking at the relative amount of collagen, the nucleus region had become more fibrous, indicating that degeneration was progressing (see Fig 9 for typical results, note that to clearly show the effect of change in loading, the results are visualized on the generic disc geometry). Similar changes but in the reverse direction was observed for the GAG content as well as the water content. Although the changes were modest, the results indicated that the biochemical content was progressing towards the next stage of degeneration. However, based on the relative change in water content inside the IVD, no grade change would be anticipated in the upper level adjacent to the treated disc within the first 2 years after surgery. Matching this result, no change in degeneration grade was observed clinically in all cases.

**Fig 10:**
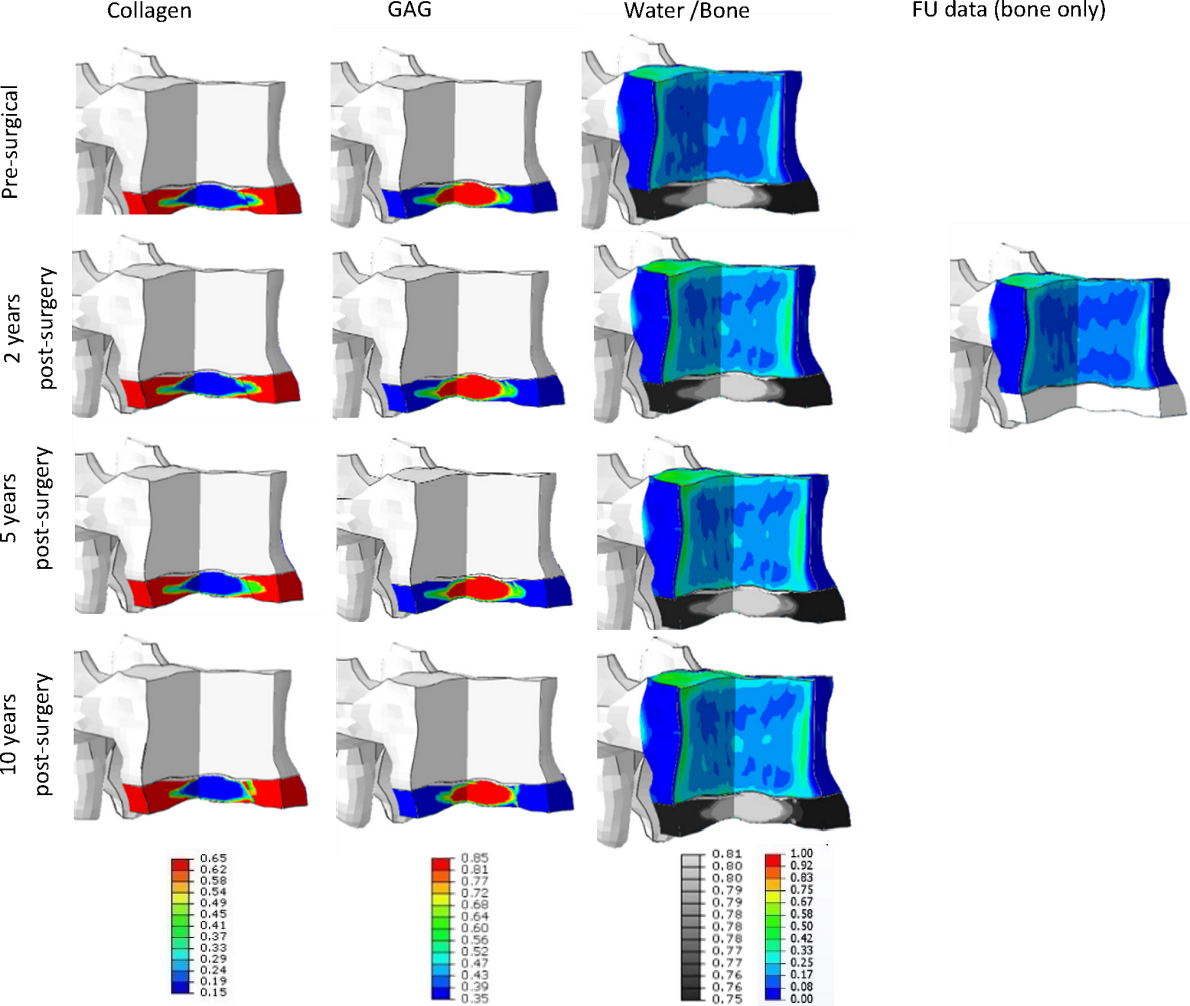
Pt-specific results up to 10y post-operative of case #4, visualised on the pt geometry. Tissue changes in both bone and disc changes simulated up till 10 years post-operative in a combined view. For disc tissue, the ECM components are visualized: collagen, GAG and water content (relative amount). Water content is visualized in black and white, mimicking the water content as could be observed on MRI data. Next to these ECM changes, also bone changes are visualized for the 4 different time points. After 2 years, the clinical FU-data is visualized to show a graphical comparison. Bone density values vary between 0 and 1, relative collagen content between 0.65 and 0.15, GAG between 0.85 and 0.35, water content between 0.81 and 0.75 respectively.

**Fig 11:**
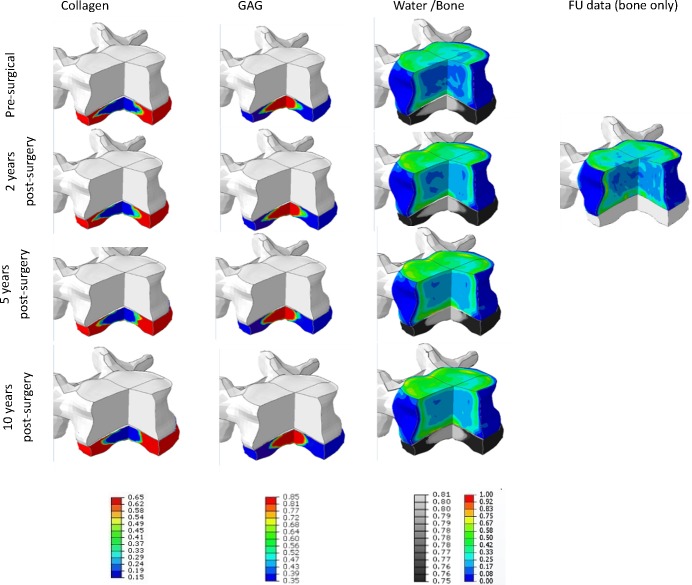
Pt-specific results up to 10y post-operative of case #6, visualised on the pt geometry. Tissue changes in both bone and disc changes simulated up till 10 years post-operative in a combined view. For disc tissue, the ECM components are visualized: collagen, GAG and water content (relative amount). Water content is visualized in black and white, mimicking the water content as could be observed on MRI data. Next to these ECM changes, also bone changes are visualized for the 4 different time points. After 2 years, the clinical FU-data is visualized to show a graphical comparison. Bone density values vary between 0 and 1, relative collagen content between 0.65 and 0.15, GAG between 0.85 and 0.35, water content between 0.81 and 0.75 respectively.

**Fig 12:**
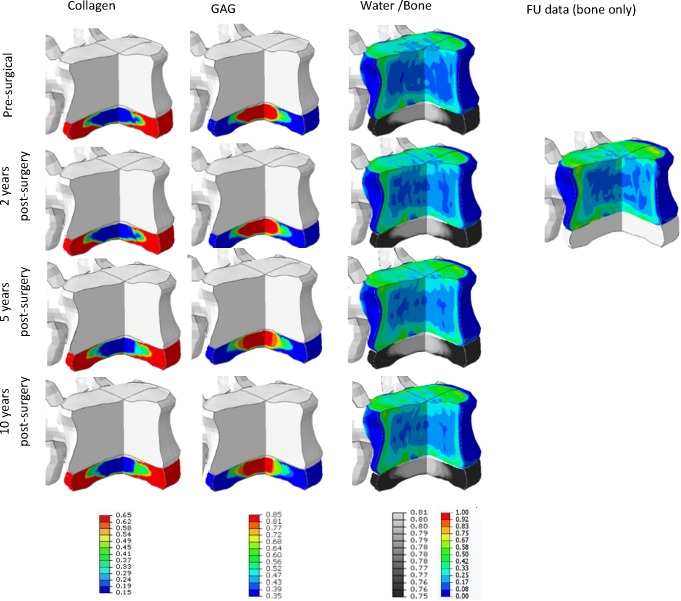
Pt-specific results up to 10y post-operative of case #9, visualised on the pt geometry. Tissue changes in both bone and disc changes simulated up till 10 years post-operative in a combined view. For disc tissue, the ECM components are visualized: collagen, GAG and water content (relative amount). Water content is visualized in black and white, mimicking the water content as could be observed on MRI data. Next to these ECM changes, also bone changes are visualized for the 4 different time points. After 2 years, the clinical FU-data is visualized to show a graphical comparison. Bone density values vary between 0 and 1, relative collagen content between 0.65 and 0.15, GAG between 0.85 and 0.35, water content between 0.81 and 0.75 respectively.

Three cases (#4, #6, #9) were simulated up till 10 years. For the first two years, the predicted tissue changes correspond well to the clinical data and only modest changes were predicted (Table 5). For case #9, after 5 years a significant change in ECM content was simulated as well as a significant change in bone density (Fig 10, Fig 11 and Fig 12). For the other two cases, only modest changes in both bone and ECM content were simulated. After 10 years, in all cases an increase in degeneration of one grade was predicted.

## Discussion

The constituent based FE model of an IVD was successfully extended to a mechanoregulated IVD model. Based on a mechanoregulated tissue differentiation theory [23], a stable healthy IVD with a realistic ECM spatial distribution was obtained. Subsequently, the IVD model was incorporated into a spinal segment model and was extended with a bone remodelling theory [28]. Finally, this method was successfully extended to a patient-specific algorithm and demonstrated for 10 cases that underwent fusion surgery to predict vertebral bone density and disc tissue adaptive changes in the adjacent segment.

In both the clinical study and the computer simulations, a modest increase in vertebral density was found as the result of a neighbouring fusion operation. As observed in multiple studies, this effect may lead to accelerated disc degeneration of the adjacent disc levels since an increase in stiffness may lead to changes in loading configuration at the disc as suggested by Pye et al. [13]. In the current study, however, the observed and simulated changes in density were small, as only a 2 year follow-up period was available. Interestingly, the simulation study predicted ECM changes towards the next degeneration grade, suggesting that a longer follow-up period would reveal more severe degeneration. This was clear also from the three cases in which simulation results were continued until 10 years follow-up, where a clear trend in disc changes were observed, especially from 5 years post-surgery, even when changes within the first 2 years were modest. These findings are in line with those found by Pye et al. [13], Aota et al. [8] and Etebar and Cahill [55] who observed a change in grade after 25 months or more.

A point that requires further discussion is the fact that the total adaptation model as presented here involves a large number of parameters, of which only a limited number (i.e. disc/vertebral geometry, disc composition, bone density distribution and changes in load between the pre- and post-operative situation) could be determined in a patient-specific manner. The remaining ones were based on literature values or determined using a fitting procedure. The solution of this fitting procedure may not be unique. For example, it was shown in earlier studies that the results for the disc are not sensitive for all parameters [22], making them difficult to fit. Similarly, for the bone remodelling simulations, different combinations of the osteocyte sensitivity μ and the osteoblast bone formation rate τ can provide the same results, and due to the specific formulation used the results are not dependent on the osteoclast resorption chance *f*_*ocl*_ and resorption volume *V*_*res*_ (when assuming these do not change pre- and post-operative). Although it would be possible to remove or lump such parameters, it was chosen not to do so here since they all have a clear physical interpretation such that reasonable estimates are possible from literature. Keeping them included may make the model more versatile in case of future improvements in patient measurements, and enable the model to account for changes that are neglected in this study (e.g. effects of bisphosphonate treatment that affect osteoclast activity). Whereas the sensitivity of the results of the disc degeneration and the bone remodelling algorithm has been investigated in earlier studies [22,28], it was not possible to address this for the combined patient-specific model used here. As such, the present study merely serves as a demonstration of the feasibility of such a combined model than as a rigorous validation. Obvious, a parameter sensitivity study would be required to better investigate the effect that individual parameters have on the outcome and thus the need for patient-specificity. The bone remodelling algorithm taken from Colloca et al., [28] was modified to account for the difference in SED values in the pre- and post-operative situation. The actual remodelling algorithm used here is similar to those used to predict bone loss around implants (e.g. Huiskes et al., [25]), and also referred to as a ‘site-specific’ remodelling. The major advantage of this approach is that realistic results can be obtained even when evaluating only a limited number of daily loading conditions and that it results in more robust predictions.

Although predicted changes in bone density show similar trends as those observed in the patients, there also are clear differences. Several reasons could explain such differences. First, the change in loading due to fusion was calculated from the full lumbar model [29] only once by comparing the pre- operative and post-operative loads. Ideally, the BCs are re-determined after each adaptation cycle since changes in disc adaptation might affect spine behaviour. However, such effects are expected to be very small. Second, the value of some remodelling parameters was determined by fitting the simulation results and the clinical results of only one patient. A more rigorous fitting involving more patients and a more advanced optimization scheme might improve the overall performance of the model. Since the present study involved only 10 patients, this was not possible. Third, a standardized loading protocol was used for all patients and it was assumed that the activity levels before and after the operation would be the same. Although the model could account for different loading patterns, e.g. related to occupational behaviour, or changes in activity level after the operation, no information was available for the patients included here. Fourth, there might be patient-specific differences in bone and disc metabolism and adaptation rates. For example, in patients on bisphosphonate treatment the changes in bone density might be less than expected. Since the model includes the cell level, it could also account for such changes in bone and disc responsiveness, provided that the effects of drugs on cell activity are known. For the present study, no information about factors that may affect the bone or disc adaptation was available.

There were also a number of technical limitations to the model used here. Most importantly, it was not possible to measure patient-specific disc composition based on the MR images. Consequently, only the Pfirrmann grade could be used to estimate the disc composition. The present study, however, was not powered to detect significant changes in Pfirrmann grade within a 2 years follow-up period. As a result, no changes in Pfirrmann grades could detected and thus it was not possible to validate the disc degeneration model relative to the patient study. As mentioned above, this limits the present study to a feasibility study. Possibly, more advanced MR techniques, such as T1rho and T2 mapping may better reveal the patient-specific disc composition.

Second, because of the lack of accurate patient-specific disc composition and because the calculation of the equilibrium situation for the disc is very time consuming, it was not possible to get an equilibrium state for the disc for each individual patient. Instead the equilibrium situation was determined only for a generalized disc geometry and a mapping procedure was used to map predicted compositional changes back to the patient model. Since the generalized model obviously lacks the specific patient geometry of disc and vertebrae, this approach cannot account for geometry-related (changes in) force distribution. It does, however, account for composition-dependent (changes in) load transfer.

Finally, in order to predict a realistic material distribution, some parameters in the mechanoregulation theory had to be modified. This was based on the assumption that the original theory did not take residual strains into account and that these strains are on the same order of magnitude as load-induced strains in IVD tissues where substantial osmotic swelling is balanced by collagen tensile strains. The accuracy of the parameters as found here is not known since no other data is available to which these can be compared. As such, they should be treated as 'effective' parameters only.

## Conclusion

In conclusion, a framework was developed and tested that predicts patient specific tissue changes adjacent to the fusion region. To the best of our knowledge, this is the first framework able to simulate pt-specific tissue changes after lumbar spinal fusion in both vertebra and disc. Both bone and disc tissue changes correlated with the clinical observed findings at 2 years post-surgery.

## Abbreviations

AF: Annulus Fibrosis
BC: Boundary Condition
BV/TV: Bone density (volume fraction)
BMD: Bone Mineral Density
CS: Chondroitin Sulfate
ECM: Extra Cellular Matrix
FCD: Fixed Charge Density
FU: Follow-Up
FE: Finite element
GAG: GlycosAminGlycans
IP: Integration Point
IVD: InterVertebral Disc
KS: Keratan Sulfate
MDC: Mean Detectable Change
MS: mechanical stimulus
MW: Molecular Weight
NP: Nucleus Pulposus
SED: Strain Energy Density
